# Root Systems Research for Bioinspired Resilient Design: A Concept Framework for Foundation and Coastal Engineering

**DOI:** 10.3389/frobt.2021.548444

**Published:** 2021-04-26

**Authors:** Elena Stachew, Thibaut Houette, Petra Gruber

**Affiliations:** ^1^ Biomimicry Research and Innovation Center BRIC, Department of Biology, The University of Akron, Akron, OH, United States; ^2^ Biomimicry Research and Innovation Center BRIC, Myers School of Art and Department of Biology, The University of Akron, Akron, OH, United States

**Keywords:** root architecture, root research, biomimicry, bioinspired design, building foundations, coastal engineering

## Abstract

The continuous increase in population and human migration to urban and coastal areas leads to the expansion of built environments over natural habitats. Current infrastructure suffers from environmental changes and their impact on ecosystem services. Foundations are static anchoring structures dependent on soil compaction, which reduces water infiltration and increases flooding. Coastal infrastructure reduces wave action and landward erosion but alters natural habitat and sediment transport. On the other hand, root systems are multifunctional, resilient, biological structures that offer promising strategies for the design of civil and coastal infrastructure, such as adaptivity, multifunctionality, self-healing, mechanical and chemical soil attachment. Therefore, the biomimetic methodology is employed to abstract root strategies of interest for the design of building foundations and coastal infrastructures that prevent soil erosion, anchor structures, penetrate soils, and provide natural habitat. The strategies are described in a literature review on root biology, then these principles are abstracted from their biological context to show their potential for engineering transfer. After a review of current and developing technologies in both application fields, the abstracted strategies are translated into conceptual designs for foundation and coastal engineering. In addition to presenting the potential of root-inspired designs for both fields, this paper also showcases the main steps of the biomimetic methodology from the study of a biological system to the development of conceptual technical designs. In this way the paper also contributes to the development of a more strategic intersection between biology and engineering and provides a framework for further research and development projects.

## Introduction

Currently, 40% of the global population lives in cities and by 2050, this number will increase to 66% (Li, 2018). 40% of the global population and 75% of the world’s megacities are within 100 km of a coastline and this percentage is also expected to increase (Mayer-Pinto et al., 2019). These population migration trends highlight the need for built infrastructure, competing for space with natural habitats that provide essential protective and regulating ecosystem services (Duraiappah et al., 2005; Lotze et al., 2006; Grimm et al., 2008). Compounded by climate change, damage to the built environment from natural disasters incurs massive economic losses (Tamura and Cao, 2010; Dinan, 2017).

Continued urban migration results in growth of infrastructure and of impermeable surface cover. The overuse of material with respect to foundation construction specifically, also increases soil compaction. Soil compaction and impermeability compromise water storage and infiltration and so contribute to increasing risks of flooding and erosion (Yang and Zhang, 2011; Alaoui et al., 2018). Soil erosion becomes a problem for foundations as their anchorage depends on soil stability. Increasing frequency and intensity of storm events will also impose more severe loading scenarios (Dinan, 2017). Reducing soil compaction, preventing erosion, and adapting to extreme loading scenarios are crucial needs, questioning the current design of building foundations. A multifunctional adaptive approach to foundation engineering should aim at alleviating flooding and erosion potential, while also lowering material usage in construction required to support a structure under various loading scenarios. As seen in the evolution of biological systems, multifunctionality typically increases design complexity. The difficulty of inserting complex structures in the soil without significant excavation in current civil engineering methods limits foundation design to simple morphologies.

Coastal infrastructure protects populations and the built environment against wave action and landward erosion (Bulleri and Chapman, 2010; McLachlan and Defeo, 2018). Continued coastal migration and the effects of climate change require more protective infrastructure that is also substantially larger in size and scale (Ferrario et al., 2014). This trend eliminates, displaces, or fragments natural coastal habitats which provide multiple significant ecosystem functions (Barbier et al., 2008; Strain et al., 2018), not to mention substantially decreasing biodiversity for some of the most diverse global ecosystems (Duarte, 2009). Additionally, traditional coastal engineering practices often cause downstream erosion, wave reflection, bottom scour and subsequent increased nearshore wave heights, and disruption of natural nearshore littoral transport (Silvester, 1972; McLachlan and Defeo, 2018). A multifunctional adaptive approach to coastal engineering should aim at wave attenuation, dissipation, and dispersion to reduce wave action and erosion potential, while also creating physical conditions, such as quiescent flow regimes and habitat refuge spaces, to increase and maintain biodiversity across multiple taxa (e.g., plants, macroinvertebrates, and fish).

We propose that the overarching design framework of biologically inspired design (BID), hereinafter referred to as bioinspired design, can inform the development of sustainable, multifunctional, and adaptive innovations to built infrastructure. Bioinspired design utilizes inspiration from nature to develop technical outcomes (Lenau et al., 2018). In our case, understanding how living organisms embed and stabilize themselves with minimal disruption and degradation to their surroundings, dynamic environment is crucial to our application areas of building foundations and coastal infrastructure. Natural ecosystems contain herbaceous vegetation, woody plants, and trees, in which roots contribute significantly to anchorage of an aboveground structure and subsequent substrate stability. In the case of mangroves and other coastal forests, their root systems must significantly contribute to wave attenuation and substrate stability along coasts for survival (Koch et al., 2009). Roots also perform multiple functions other than anchorage and substrate stability and adapt to changes detected in the surrounding soil environment through a variety of mechanisms (Malamy, 2005). Therefore, we hypothesize that the study of root systems informs multiple engineering design applications in the areas of foundation and coastal engineering.

Within the framework of bioinspired design lies both biomimetics and biomimicry (Lenau et al., 2018). For the scope of this work we utilize the terms synonymously and employ primarily the problem-driven process of biologically inspired design as our research methodology to present design proposals for our specific application areas. Problem driven biologically inspired design takes on a technical question that is answered by a strategic search for analogous solution in biology. The first step in the problem-driven bioinspired design process and in our research investigation is an assessment of common practices, uses, and applications to identify the technical shortcomings of current building foundation and coastal infrastructure designs. Next, these shortcomings are abstracted, so that the problem, its context, constraints, and necessary functions can be transposed to biology and connected to biological analogs. Principles are extracted from biological models (in our case, root systems) out of their natural context, so that they may be emulated in technological solutions (Vincent et al., 2006; Fayemi et al., 2017). While biomimicry primarily follows the same design steps as biomimetics, its unique attribute is on an ecological philosophy and ethos to meet the challenges of sustainable development (Benyus, 2011; Lenau et al., 2018).

To demonstrate the hypothesis that the study of root systems informs multiple engineering design applications through the overarching design lens of bioinspired design, we present an overview of relevant root biology in “Roots as Biological Model” section, with a special focus on adaptation and biomechanics. Through the biomimetics process, specific biological information is then related to infrastructure problems and vulnerabilities through a functional translation in a comprehensive analogy table in “Abstraction and Analogy” section (Table 1). “**Application of Root Biology to Technical Designs**” section presents a range of current and future innovative bioinspired design concepts for the fields of building foundation and coastal engineering, followed by Discussion and Conclusion in sections “Discussion” and “Conclusion”.

**TABLE 1:** Analogy table (“Abstraction and Analogy” section).

		Biological role models	Functions/Working principles	Problems/vulnerabilities
Soil erosion	1	Root/soil plate network behaving as one entity due to adhesion between soil particles and presence of root hairs Coutts (1983); Bailey et al. (2002)	Network of thread-like elements in contact with granular media to distribute load prevents movement of this media in response to tensile and shear forces	Soil erosion around building foundations; for example, during heavy precipitation events, or exposed location on a steep slope/cliff (with or without precipitation)
	2	Single root fan facing upstream deflects flow, additionally disrupts, partitions and slows the flow that passes through fan via drag, resulting in less scour within the structure Svoboda and Russell (2011)	Single flow deflection structure oriented in direction of predominant flow, composed of cylindrical elements with variable length, cross section, diameter/width, orientation and curvature arranged in a non-uniform porous branching pattern that disrupts flow through structure	High water velocity leading to erosion and poor habitat conditions
	3	Position and orientation of several tightly placed rootwads in naturally occurring, stable log jams, including those constructed by beaver for habitat Abbe et al. (1997); Abbe and Montgomery (2003); Svoboda and Russell (2011)	Large cylindrical elements with complex fractal-like endings facing the flow act as key anchoring and stabilizing elements of a single assembled porous yet stable structure of multiple elements	Coastal erosion and scour, specifically caused by wave action and reflection
	4	Irregular distribution, configuration and porosity of roots and tree trunks in mangrove swamps resulting in flow obstruction/wave attenuation Mazda et al. (1997); Kazemi et al. (2017)	Semi-rigid elements in a varied distribution of spacing and orientation in a continuous and connected system causing wave attenuation with reduced reflection; also increasing drag, which reduces downstream flow velocity and shear stress	High velocities and wave action in nearshore area leading to coastal erosion, turbidity, poor habitat conditions due to high water flow and poor water quality, and inland flooding risk
Structural support	5	Root system architecture recruiting large volume of soil and surface area to support tree and respond to variable loading conditions	Structural support through a wider distributed network of elements	Low resilience of foundation piles to changing loading conditions due to limited volume of soil used for support due to simple shape
	6	Interweaving of roots and root grafting between trees of same species contributing to mechanical support Graham and Bormann (1966); Kumar et al. (1985); Keeley (1988)	Continuous weaving of thread and stem like elements into a connected network in granular media	New engineering structures not connected to or benefiting from existing artificial structures already in place
	7	Asymmetric root morphology resisting asymmetric loading conditions due to wind and weight of tree canopy Young and Perkocha (1994); Nicoll and Ray (1996); Nicoll and Dunn (2000); Tamasi et al. (2005)	Structural adaptation under asymmetrical load by increasing number of rigid elements on the compression side and thread-like elements on the tension side	Engineering structures not designed to support and adapt to specific directional loading conditions
	8	Differentiated root morphology for sloped terrain Reubens et al. (2007); Danjon et al. (2008); Stokes et al. (2009); Liang et al. (2017)	Main deep sinker element providing anchorage with shallow thread-like elements retaining soil particles in a sloped terrain to stabilize structure and media	Engineering structures—such as foundations and coastal infrastructure—lacking specialized adaptation or design for sloped terrain
	9	Adapted root distribution to chemical and mechanical soil conditions Ennos (2000)	Adaptation of structural morphology to changing environment	Fixed engineering structures unable to change/adapt to changing environment
	10	Mangrove root morphology supporting and erating the tree in both low-tide (roots surrounded by air) and high tide (roots surrounded by water) environments Ohira et al. (2013); Hogarth (2015)	Flexible branching/network able to transfer varying loads to granular media when surrounded by fluid of different densities	Structures built for one water level not effective outside of their designed range (e.g., seawall height unable to counter sea level rise)
	11	Buttresses transferring loads from the trunk to the soil/root plate Young and Perkocha (1994); Crook et al. (1997)	Element connection shape optimized for stress reduction based on the tension triangles rule Mattheck et al. (2006)	Stress concentrations in connections
	12	Development of a "T" or "I" cross section in structural roots Nicoll and Ray (1996); Nicoll (2006)	Adaptation of the element's cross-sectional profile in response to specific loading conditions	Fixed cross section of elements, overdesigned to resist diverse loading conditions
	13	Design of lateral roots and root hairs that physically attach to soil particles at the micro scale Bailey et al. (2002)	Increase loading capacity of macro structures through skin frictional contact between granular media and network of thread-like elements by integrating highly textured micro surfaces	Foundations designed at macro scale not utilizing micro interactions between foundation and soil particles to increase loading capacity
	14	Root mucilage enhancing bond strength between soil particles and roots to counteract soil shrinkage/expansion caused by rapid wetting/drying cycles Czarnes et al. (2000); Galloway et al. (2020)	Increase loading capacity of macro structures by attaching thread-like elements to granular media with chemical adhesion	Foundations not chemically connected to the soil particles at the micro scale for increased loading capacity
Soil penetration	15	Cone shaped root morphology due to growth resulting in diameter gradient along root axis from thin root tip (earliest growth) to thick root flair (mature growth)	Tapered element to facilitate penetration of granular media	Mechanical resistance of soil overcome with higher forces to penetrate soil
	16	Contractile root behavior pulling the plant into the soil to protect plant organs from extreme temperature, low moisture and increase anchorage North et al. (2008)	Creating a shortening of the attachment to lower attached element to reduce exposure to extreme conditions, also increases tensile force and improves anchoring	Engineering structures degrading over time under weathering and tensile structures yielding under constant loading
	17	Root turning in the soil by differential growth response, triggered by auxin distribution in the elongation zone Chen et al. (1999); Blancaflor and Masson (2003)	Turning in a granular media by differential expansion of a thread-like element	Inability to change direction of soil penetration in granular media when driving foundation piles into soil, mostly vertical or near-vertical orientation
	18	Root hairs and root curvature anchoring the root allowing the root tip to move forward in the soil due to cell elongation Bengough et al. (2011)	Combination of functions: Anchorage and size expansion from anchoring point, therefore resulting in forward movement	Construction equipment limited to pushing and expanding a structure just from the surface through the soil
	19	Circumnutations of root tip to find path of least resistance in the soil to facilitate growth Minorsky (2003); Migliaccio et al. (2013)	Moving the tip of the digging element in a circular or spiral path to find least resistance regions in granular media	Difficulty of finding path of least resistance when digging or pushing through granular media
Conditions for living organisms	20	Space between mangrove roots differing with respect to height Twilley and Day (2013)	Distribution and geometry of voids with respect to organism body size supporting habitats for organisms, diverse predator-prey interactions and prey refuge	Lack of habitat complexity along hardened shorelines reducing diverse food web interactions
	21	Snag/root roughness preferred substrate for invertebrate colonization, increasing foraging habitat for prey fish Angermeier and Karr (1984); Wallace and Benke (1984); Benke et al. (1985)	Heterogeneous surface textures and structures	Hard, flat and smooth surfaces of coastal infrastructure reducing habitat availability for sessile or habitat-forming organisms
Multifunctionality	22	Thermal energy absorption from the soil by roots and distribution to the tree Ballard et al. (2009)	Utilizing stable temperature of soil to heat/cool a system	Current building foundations not designed to actively contribute to geothermal exchange in buildings
	23	Mangrove root adaptation in anaerobic, high salinity, waterlogged soils Robertson and Alongi (1992); Hogarth (2015)	System able to develop in harsh environment due to adaptive survival strategies that creates favorable environment for other systems to function and exchange resources	Static, heavy and bulky structures required to provide stability of waterlogged muddy soils eliminating space for natural habitat
	24	Root system and soil exchange of nutrients, carbon and water, also between mycorrhizal fungi when present	Constant exchange of resources with the environment to enhance growth and adaptation to stimuli	Engineering structures unable to facilitate exchange of water and resources with the soil (e.g., water uptake, water discharge, carbon sequestration)
	25	Self-healing properties of trees by accretional growth around wounds Bloch (1952); Cremaldi and Bhushan (2018)	Adaptive gap closure through material accretion	Engineering structures—such as foundations - often inaccessible for active repair

## Roots as Biological Model

Rather than a comprehensive encyclopedia this section provides a general overview of root biology and an understanding of strategies and mechanisms found in root systems for mechanical anchorage, soil stability, and other dynamic external loading conditions relevant for biomimetic translation to the two application spaces of building foundation and coastal infrastructure design. Additionally, there is a general introduction to the use of root systems (and other woody components) in natural constructions by humans, whose strategies and mechanisms of construction are also relevant to biomimetic translation.

### Root Basics

#### Root Structure Components

Root structure is generally described by four regions or zones (Bidlack et al., 2011). These regions, starting from the end of the root, are the root cap, region of cell division, region of elongation, and region of maturation. The root cap and apical meristem located in the region of cell division are the only regions that push through the soil. The other regions remain stationary. Root diameter gradually increases through addition of secondary tissues in the region of elongation (i.e., radial growth). Lastly, the region of maturation is where root hairs are produced. These are short-lived extensions that adhere tightly to soil particles and increase the total water and mineral nutrient absorptive surface of the root.

#### Root Classification: Different Types of Roots

There are three main types of roots: primary (i.e., seminal), adventitious (i.e., nodal), and lateral roots (Malamy, 2005). Primary roots stem from seed, while nodal roots initiate from non-root tissue and are coordinated with aboveground shoot development. Many mature plants have a combination of taproot (a thick, vertical, centrally located primary root) and diffuse fibrous (i.e., nodal) root systems (Malamy, 2005; Bidlack et al., 2011). Lateral roots develop by branching, which is coordinated with root elongation (Lecompte and Pagès, 2007), with an equilibrium maintained between root number and length (Malamy, 2005). From a spatial perspective, structural “coarse” roots (sometimes referred to as basal roots) are often near the base of the stem. Their primary function is anchorage, and they may develop considerable secondary thickening. Fine “thin” roots are often much further away from the stem (sometimes referred to as distal roots). Their primary functions are soil exploration to source water and nutrients.

#### Root Growth Processes

Axial growth and radial growth are the two main types of root growth processes (Hodge et al., 2009). Axial growth is defined as the root extending in length and the tip pushing forward into the soil, with the root parts behind the elongation zone anchored in the soil. The direction of root elongation is triggered by different tropisms, such as gravitropism and hydrotropism (Lynch and Brown, 2001). Axial growth is significantly limited when zones with high soil mechanical resistance is present (Hoad et al., 2001).

Radial growth is defined as additional layers of growth on individual roots, root thickening, or secondary thickening (Hodge et al., 2009). This growth process is important in expanding the range of root functions, including axial transport properties, mechanical strength and anchorage, storage capacity, and protection against predation, drought, or pathogens.

#### Root System Architecture and Morphology

Root System Architecture, or spatial configuration of the root system, varies greatly depending on plant species, soil composition, water, nutrient, and mineral availability (Malamy, 2005; Hodge et al., 2009). The shape of a root system is characterized by how the roots occupy the soil and is defined specifically by the traits of root depth, lateral root expansion, and root length densities. The shape of the root system can also be described by abstract synthetic descriptors like fractal dimensions (Tatsumi et al., 1989). The structure is characterized by root system components and their relationships, defined by the traits of root gradients, cross section, topology, and connection between roots (i.e., branching angle) (Malamy, 2005). Root topology describes the abstracted pattern of root branching. Topological order is an important parameter of root trait analysis as it can be a stronger predictor of mechanical properties than root diameter (Mao et al., 2018).

There are three main categories of root system morphology (Ennos, 2000). The plate morphology, often found in mature trees, is characterized by thick lateral roots radiating horizontally or slightly obliquely from the main stem, followed by tapering and branching, in addition to sinker roots originating from lateral roots close to the stem. The taproot morphology, characterized by the single, centrally located taproot, is often found in dicotyledon species (Ennos and Fitter, 1992) and some rainforest pioneer species (Crook et al., 1997). Coronal and prop root morphology is often found in monocotyledon species, as they cannot undergo radial growth and therefore cannot produce a taproot. This type is characterized by thick lignified nodal roots growing obliquely from the stem (Ennos, 1991; Ennos et al., 1993). Many species possess intermediate morphologies (Crook and Ennos, 1998). Intraspecific root grafting seen in forests is believed to contribute to mechanical support and nutrient exchange (Graham and Bormann, 1966; Kumar et al., 1985; Keeley, 1988). Additionally, root system morphology can be affected by symbiotic root—microorganism relationships in the rhizosphere, such as mycorrhizal fungi and actinomycete bacteria (Steeves and Sussex, 1989; Hodge et al., 2009).

### Root Function, Development, and Adaptation

#### Root Adaptation to Soil Patches

To effectively deploy in transient soil patches rich in moisture or nutrients, roots exhibit significant morphological plasticity through modular root structure and tissue differentiation along the root axis (Hodge et al., 2009). Drew and Saker (1975) reported an increase in lateral root initiation in soil patches, while Linkohr et al. (2002) found a repression of lateral root elongation outside the patches. Root systems also shed roots when resource uptake becomes insufficient (Hodge et al., 2009).

#### Root Adaptation to Soil Density, Compaction, Resistance, and Moisture

Roots must overcome soil resistance to displace soil particles as the root grows. As a result, root diameter increases and root elongation decreases with increasing soil strength (Correa et al., 2019). Soil zones of variable resistance impact root growth rate, morphology, orientation, and the local soil-root environment (Hodge et al., 2009 and associated references therein). Roots generally follow the path of least resistance, leading to distinct environments compared to the bulk soil (Pierret et al., 1999; Hodge et al., 2009).

To grow through soils, root tips need to generate enough force to expand a hole in the soil, exceed frictional resistance of the root tip with the soil particles, and exceed the internal tension in the root cell walls (Bengough et al., 2011). It is suggested that up to 80% of total penetration resistance results from friction (Greacen et al., 1968; Bengough et al., 1997). The friction between soil particles and roots, presence of root hairs, and potential root trajectory also assist in anchoring the root, so that tissues in the elongation zone can push the root tip forward (Bengough et al., 2011).

Circumnutations (i.e., revolving nutation), present in all plant organs (Hart, 1990; Kiss, 2006; Mugnai et al., 2007), are the result of differential growth, resulting in active growth movement following an elliptical path in a left-handed or right-handed rotation (Johnsson, 1997). The role of root circumnutations is still debated, but Dottore et al. (2018) found that this movement reduces the pressure and energy required to penetrate soil.

Roots passively secrete low molecular weight organic compounds in the rhizosphere, called root exudates. These exudates promote microbial activity and soil stabilization through mucus like adhesion, known as mucilage (Tisdall et al., 1978; Cheshire, 1979; Amellal et al., 1998). Rapid wetting/drying cycles induce shrinkage and cracks in the soil, which reduces hydraulic conductivity due to the presence of large pores in the soil matrix (Grant and Dexter, 1989). Czarnes et al. (2000) found that a root mucilage analog (e.g., polygalacturonic acid) stabilized the soil structure against the disruptive effects of wetting/drying cycles.

#### Root Adaptation to Continual Water Inundation and High Salinity

Flooding induces ethylene production in the root, which signals increased nodal and lateral root formation posited for increased stability (Hodge et al., 2009). Mangroves, exhibiting a complex stilted root network, only exist in tropical climates in cyclically submerged environments, with muddy, waterlogged anoxic soils and high salinity. Mangrove roots can generally be classified into four types: stilt root, knee root, snorkel root, and buttress root (Tomlinson, 2016). To obtain adequate oxygen supply from the air to belowground roots, mangroves increase adventitious root production specifically with spongy, erenchymous tissue near the sediment surface (Hodge et al., 2009). Pneumatophores, vertical erect roots that emerge from shallow adventitious roots (Bidlack et al., 2011), are known to slow water currents, attenuate waves, and increase sedimentation (Mazda et al., 1997; Hogarth, 2015).

#### Contractile Roots Adapted to Environments With Low Water Availability

Contractile roots are found across multiple plant groups, which mostly inhabit environments with harsh seasons such as drought or cold temperatures (Pütz, 2002). This behavior, which protects plant organs and young shoots from harsh conditions by pulling them down into the soil, is also known to improve plant anchorage and water uptake (Jernstedt, 1984; Pütz, 2002; North et al., 2008; Bidlack et al., 2011).

### Root Biomechanics

#### The Root-Soil Plate: Effects of Behavior as One Mechanical Entity

In Coutts (1983) and associated references therein, various studies on the behavior of rooted soil under stress found that tree roots increased soil shear strength by 1–17 kPa. When resistance of the root-soil interface is higher than the surrounding soil strength, the root-soil mass behaves as a single unit under applied load, known as the root-soil plate. This plate is especially visible in uprooted trees. Root-soil resistance is affected by branching and distribution in number and size of roots. Roots stiffen the soil similarly to how rebar rods stiffen a beam, as they mostly resist tensile loads. Depending on soil conditions, root breakage and slippage through soil are the main failure mechanisms. In clay soils where the soil resistance is greater, roots slip instead of break, meaning that root-soil resistance is more a function of soil resistance than root morphology and strength (Waldron, 1977). Root morphology and strength play a greater role when the soil moisture content is a little below its saturation point.

#### Effect of Root Hairs on Anchorage and Growth

Ennos (1989) suggests in a study on sunflowers that root hairs play a major role for the anchorage of young plants against uprooting by increasing the effective root surface area in contact with the soil. Additionally, Stolzy and Barley (1968) saw an increase in tension resistance of individual roots of *Pisum sativum* seedlings with root hairs compared to ones without root hairs. According to Ennos (2000), it is far less likely that root hairs are useful in the anchorage of mature plants, since root hairs are only produced near the tip of elongating roots in the maturation zone where mechanical stresses are relatively low for large mature plants. In this case, the major mechanical role of root hairs is in root tip growth, as root hairs anchor the root while the tip is pushed forward through the soil (Stolzy and Barley, 1968; Ennos, 2000; Bengough et al., 2011).

#### Effect of Roots on Slope Stability

Trees reduce soil erosion and prevent shallow landslides through a network of coarse and fine roots just below the surface that increase the shear strength of the soil medium, and sinker roots that anchor the surface layers to a deeper, more stable soil mass (Nicoll et al., 2005). Structural root mass has been found to be greater on the upslope side of exposed trees on slopes, explaining the increase in resistance to upslope overturning (Nicoll and Ray, 1996). Liang et al. (2017) demonstrated in a slope stability simulation using a 3D printed root structure that root strengthening pushes the soil shear plane deeper in the soil. Root strengthening depends on species-specific root mechanical properties, surrounding confining stress, depth of the initial soil slip plane, and root morphology. The maximum reinforcing effect from root strengthening may require increased root depth of sinker roots and lateral extension to enhance soil shear strength. In a Fiber Bundle Model (FBM) framework to estimate root cohesion, Arnone et al. (2016) found that the effects of root water uptake may be more significant than mechanical reinforcement for slope stability, especially in fine soils.

#### Effect of Roots on Wave Attenuation

In a mature mangrove forest, Mazda et al. (1997) observed that wave attenuation does not decrease with increasing water depth, which is very beneficial in cases of storm surge and sea level rise. Mangrove swamps of *Rhizophora spp.* or *Bruguiera spp.* have intricate and large pneumatophores and therefore provide resistance to flow due to increased projection area. Wave energy loss is caused by both bottom friction and flow resistance (i.e., drag force) by mangrove vegetation (trees, trunks, and roots) through the entire water column. The submerged tree volume and projection area of aboveground mangrove root morphology play a significant role in attenuating tsunami inundation flow (Ohira et al., 2013).

#### Effect of Roots to Lateral Aboveground Stresses

The components and relevant parameters of anchorage under lateral forces (e.g., wind) include root-soil plate dimension, root and soil tensile strength beneath the plate, root-soil resistance specifically on the windward side, and stiffness at the pivot point at the base of the tree (Coutts, 1983). A root system of a tree subjected to wind loads responds through increased growth of the roots aligned with the plane of stimulation (Nicoll and Dunn, 2000). On the leeward side, bending and compressive forces push the root-soil interface against the soil below. On the windward side, tensile and/or shear forces are present due to uplifting.

A study conducted by Tamasi et al. (2005) showed that wind loading on young *Quercus robur L*. trees resulted in increased total lateral root number and length in wind stressed trees compared to control trees. Wind loading appears to result in increased growth of more lateral roots and higher structural root mass on the leeward side. Root systems of adult *Picea sitchensis* trees exposed to a natural prevailing wind had higher structural root mass on the leeward side instead of the windward side (Nicoll and Ray, 1996). A study conducted by Stokes et al. (1995, 1997) on young *Picea sitchensis*, showed greater numbers of both windward and leeward roots, more elongated and branched morphology, and increased root diameter.

Although tap roots play a role in initial tree anchorage (Crook et al., 1997), evidence suggests that lateral roots are the major component of anchorage in response to dynamic loading conditions (Ennos et al., 1993; Stokes et al., 1995; Ennos, 2000; Stokes, 2002; Dupuy et al., 2003; Cucchi et al., 2004). If there are too many roots in the soil however, the soil will likely fail in shear and tension at the edge of the soil-root plate (Ennos et al., 1993). Ennos (2000) also notes that plants minimize the total energy cost of anchorage when exposed to uprooting potential by only strengthening (i.e., thickening) the basal parts of the root system.

The location of roots defines their cross-sectional shape. Bending resistance seems to occur through changes in structural roots cross-sections, producing I-beam, T-beam, and oval cross-sections (Rigg and Harrar, 1931). Ennos (2000) describes the components of root system morphology that resist lateral stresses. The plate morphology has three components of anchorage: resistance of leeward hinge to bending, resistance of the windward roots to uprooting, and weight of the root-soil plate. The taproot morphology has two components of anchorage: soil compressive resistance and taproot bending resistance. The morphology of coronal and prop roots also have two components: soil compressive resistance and buckling resistance of the windward roots.

#### Effect of Buttress Roots to Lateral Aboveground Stresses

Uneven secondary thickening between root and stem results in the development of supporting buttresses (Bidlack et al., 2011). Crook et al. (1997) studied the anchorage of taproot systems: buttressed trees of *Aglaia* and *Nephelium* possessing sinker roots, and non-buttressed *Mallotus wrayi* trees with thin lateral roots. Buttresses provided six times more anchorage than the thin lateral roots of non-buttressed trees and approximately 60% of the anchorage acting in tension and compression. Buttresses of tropical trees are also more often found on the less dense side of an asymmetric crown, suggesting that buttresses partly serve as tension elements to equalize mechanical stresses (Young and Perkocha, 1994; Crook and Ennos, 1998). In addition, buttresses are believed to reduce the risk of buckling failure (Young and Perkocha, 1994), and reduce bending and concentration of stress at the base of the tree (Mattheck et al., 1993).

### Root Utilization in Human Constructions

Tree root systems have been directly utilized in several natural constructions by humans. These constructions are an example of bio-utilization or biotechnology, and in combination with traditional engineered or technical components, can take on the form of bio-hybrid approaches. In the case of streambank stabilization and restoration ecology practices using large woody debris (LWD—e.g., fallen trees, stumps, rootwads, and branches) (Svoboda and Russell, 2011), their biological analogs are in beaver dams and complexes (Wright et al., 2002), natural woody debris (WD), and natural log jams (Larson et al., 2001). Naturally occurring LWD jams were removed from many rivers for flood control and navigation during the 20th century (Montgomery et al., 2003), but these structures are currently being re-introduced due to benefits such as habitat complexity and restoration, debris retention, in addition to erosion protection, stabilization, and grading control (USBR and ERDC, 2016).

Abbe et al. (1993) studied the distinctive patterns exhibited by natural LWD jams, identifying categories and types of accumulations and jams by size, position, orientation, frequency, and type of WD. Continued in the study by Abbe and Montgomery (2003), this categorization provides a framework and typological basis for which to describe the ways these jams influence stream geomorphology, floodplain formation, and riparian habitat. Different LWD configurations and jams produce erosional and depositional zones at varying lengths downstream of the structure and/or within the structure depending on hydraulic and geomorphic project objectives. These jams can also be designed to freely move during higher velocity flows or persist for centuries as stable structures (Svoboda and Russell, 2011). The position of logs within a stream channel, wood density, and decay rates as a function of tree species and moisture content, all affect the structure’s stability and life expectancy, but will commonly exceed the design life of most engineering projects (Abbe et al., 1997).

LWD drag depends on the cross-sectional area of a flow obstruction, incident flow velocity, an “obstruction form descriptor” coefficient, and a blockage coefficient equal to the ratio of the structure’s total cross-sectional area to the channel cross-sectional area perpendicular to flow (Gippel et al., 1992; Abbe and Montgomery, 1996; Abbe et al., 1997). Similar geometrical parameters governing drag are also seen in a study conducted to examine the flow-structure interactions of modeled mangrove circular patches (Kazemi et al., 2017). Porosity, defined in the study as the ratio of submerged root volume to total defined volume, spacing ratio between cylindrical models of mangrove roots, and flexibility are influencing parameters for drag and mean downstream velocity.

## Abstraction and Analogy

Based on the review of root biology and current problem areas, an analogy table (Table 1) was created to link relevant biological principles with technical problems or vulnerabilities in the civil and coastal engineering fields via an identified abstracted function and working principle. The table can be read from both sides: starting with root biology, it allows for linking the working principle to an engineering field and starting with the technical problem area, it allows for linking to a working principle also present in biology. The main themes (i.e., soil erosion, structural support, soil penetration, conditions for living organisms, and multifunctionality), point to broader problem areas. This table provides an overview of the translation opportunities that were found from investigating both biology and engineering through the lens of biomimetics.

## Application of Root Biology to Technical Designs

In this section, we present the two main application areas for the problem-driven biomimetic approach, building foundations and coastal infrastructure. We discuss current practices, limitations and shortcomings, followed by a broad listing of current experimental and innovative solutions. We end by exploring a range of speculative, bioinspired design concepts informed by root biology to illustrate the biomimetic approach.

### Building Foundations

#### Current Foundation Design and Problem Review

Building foundations transfer aboveground structural stresses to the underlying soil by transmitting gravitational loads, stabilizing the structure against overturning and lateral movement, and providing resistance to uplift. Current foundations function by creating a contact surface area with the soil bearing the loads, pre-consolidating the underlying soil, utilizing the foundation weight under gravity, and/or anchoring it to a rock layer (Hobst and Zajíc, 1983).

Depending on soil conditions and loading scenario, foundation design follows two main types: shallow and deep foundations. Shallow foundations, such as strip footing, spread footing, or raft, transfer loads to the soil close to the soil surface and are used for low loading capacities. Deep foundations, such as piles, drilled shafts, and caissons, are used for high intensity heavy building types and resist lateral and uplifting forces. They are also used when the upper layers of soil are weak, collapsible, expansive, or subject to soil erosion (Das, 2007). They can reach depths of hundreds of meters into the ground (Frost et al., 2017).

The structural capacity of foundation piles depends on the bearing capacity of the pile tip and lateral friction of the pile (Das, 2007). Foundation pile design is determined by loading type, subsoil conditions, and location of the water table. In weak soils, point bearing piles are built up to the rock surface or into a strong soil layer if within reasonable depth. Otherwise, piles relying on friction with the soil particles or increased soil compaction are placed. In clayey soils, adhesion also helps to hold the pile in place (Das, 2007). Vibro-compaction and vibro-replacement methods are economical and well-established techniques to improve weak or loose soils through compaction (Baumann and Bauer, 1974). Depending on pile design and material, different techniques are used to insert them in the ground. Piles are driven in the soil with various types of hammers or vibratory drivers, but other techniques may be employed for specific scenarios such as jetting and partial augering (Das, 2007).

Typical foundation piles are made of wood (e.g., timber piles), concrete (e.g., precast or cast-in-situ piles), and steel (e.g., pipes or rolled H-section piles) (Das, 2007). Steel piles are easily managed, supporting high driving stresses, penetrating hard soil layers, and carrying relatively high loads. They are expensive, subject to corrosion, and may be damaged during soil insertion. Precast concrete piles also support high driving stresses while resisting corrosion, but they are more difficult to maneuver and properly cut. Cast-in-situ piles are cheaper, and the steel cast can be inspected before pouring the concrete, but the casing may be damaged during soil insertion and the resulting pile can be difficult to connect after pouring. Timber piles are limited in terms of driving force and loading conditions (i.e., capacity and direction). Composite piles are composed of different materials which are difficult to join, so they are not widely used (Das, 2007).

There are several limitations and shortcomings to current foundation design, engineering, and construction practices. First, deep foundations are limited to simple vertical or near vertical (i.e., 0° with respect to the pile axis) cylindrical piles, due to the inability of current drill and dig construction techniques to actively change direction in the soil (Frost et al., 2017). It has been demonstrated that increasing the angle of foundation piles from 0° to 15° and 30° increases the loading capacity of the foundation due to a larger bearing area (i.e., surface area of the soil in contact with the pile and supporting pile weight) (Frost et al., 2017). Compared to a traditional smooth vertical pile, the introduction of a branching angle of 15° doubled the downward bearing capacity, and a branching angle of 30° tripled this capacity (Frost et al., 2017). Additionally, orchard tree root systems showed an increase of vertical pullout resistance by 8–13 times compared to traditional micropile foundations of comparable volume and mass (Burrall et al., 2020). Second, foundations are monofunctional as they are only designed to support a structure, while we use other artificial subsurface technical structures for other functions (e.g., energy conversion). Third, the capacity of foundations to resist loads and forces is not dynamic and adaptable (some exceptional technologies exist for earthquake prone applications). Foundations are usually built as static structures and are expected to maintain morphology and materiality over time. They cannot adapt to changing environmental conditions, such as varying loads applied to the structure and soil movement, therefore operating on a single timescale. Lastly, foundations are located underground, therefore inaccessible for maintenance. The use of materials that lack self-healing properties requires an over-design to counter this potential drift in performance over time.

Root systems can serve as inspiration as they share similar functionality and design requirements with foundations such as anchorage and soil penetration, but also provide adaptability and multifunctionality. Root systems possess a large bearing area compared to their volume, due to their branched morphology and the presence of microstructures. Complex root morphology is also a result of the multiple functions provided for the tree such as soil exploration, nutrient/water exchange and transport, anchorage, and thermal regulation, which in turn provides additional ecosystem services such as erosion prevention. Additionally, root systems adapt and respond to stimuli over multiple timescales (e.g., daily fluctuations and constant long-term loads) through transient (e.g., damping) and permanent responses (e.g., reaction wood growth, self-healing). Since root systems are part of a living multicellular organism, they can heal and regenerate tissues of their anatomy. The foundation designs of the future could mimic these root system strategies.

With this biomimetic approach to foundation design, multiple research questions arise: Which design parameters informed by root systems would increase the loading capacity of foundations without adding weight? How can complex branched structures be inserted in soil with minimal disturbance? How can a foundation actively or passively adapt to changing external internal loads as informed by root adaptation mechanisms? Which additional functions could be fulfilled by foundations other than anchoring a building in place? How could biological organisms be integrated into bio-hybrid foundation designs and to what benefit?

#### Current Innovative Solutions for Foundation Design

In the following list, we summarize current innovative strategies for future foundation designs, from morphological variation to integration of biological organisms. They are organized under four main topics of interest referring to the analogy table in “Abstraction and Analogy” section (Table 1): soil erosion, structural support, soil penetration, and self-healing, as an aspect of multifunctionality.

- Preventing soil erosion—Various geosynthetic products are available on the market. The stabilizing effect of a thread-like element in granular media has been investigated by the placement of a textile filament layer by layer around loose rocks and exposed at the Chicago Architecture Biennial 2015.^1^ Additionally, bacteria that bind to soil particles, have been used to strengthen the mechanical properties of soil through Microbial Induced Calcium Carbonate Precipitation (MICP) (DeJong et al., 2006; Whiffin et al., 2007; Van Wijngaarden et al., 2011). The use of genetically modified bacteria to precipitate calcium carbonate when soil pressure is detected to react to changing loading conditions is tested with computational models (Dade-Robertson et al., 2018).
- Geometric alternatives to support structures—Foundation geometry is a defining factor for total loading capacity and pile displacement (Frost et al., 2017). Conical piles provide an increased bearing capacity compared to straight-sided cylindrical piles (Manandhar and Yasufuku, 2012). The lateral surface texture of foundation piles is another parameter to increase loading capacity by increasing shear strength of its interface with soil (Martinez and Frost, 2017). Research in this field stressed the need to design foundation surface roughness, in opposition to current smooth or only randomly structured construction materials, such as randomly textured high-density polyethylene geomembranes and roughly finished concrete (Frost et al., 2002). Biological textures, such as snakeskin, were studied to produce bioinspired surfaces designed for foundation piles and yielded promising results for increasing directional friction (Martinez et al., 2018; Martinez et al., 2019).
- Robots for soil penetration—Due to the difficulty of inserting non-linear structures in soils, burrowing robots inspired by animal (Calderón et al., 2016; Khosravi et al., 2018; Calderón et al., 2019) and plant strategies (Sadeghi et al., 2014; Hawkes et al., 2017; Sadeghi et al., 2017; Dottore et al., 2018; Greer et al., 2019; Ozkan-Aydin et al., 2019) have been explored in the past decade. For example, an earthworm inspired robot mimicking peristaltic waves by activating axial and radial contraction, was built with three silicone body segments and able to crawl through straight and curved pipes (Calderón et al., 2016; 2019). While current soil monitoring techniques use probes that are pushed in the ground, self-burrowing probes, based on radial expansion of sections of the probe, have been studied and simulated in sandy soils (Khosravi et al., 2018). Animals use their musculature to move and dig in soils whereas the root tip of plants grows through the substrate (Sadeghi et al., 2014). Root systems, and especially root tip growth, have served as inspiration for growing robots (Del Dottore et al., 2018). For example, root tip growth has been translated into a robot that can sense its environment and grow in diverse directions through additive manufacturing (Sadeghi et al., 2014; Sadeghi et al., 2017). Directional growth by extension of the body tip has also been translated in soft robotics to conform to constrained environments (Hawkes et al., 2017). In addition, the influence of circumnutation to facilitate soil penetration has been tested with artificial probe tips (Dottore et al., 2018). These movements have also been implemented in a soft robot growing in a 2D environment (Ozkan-Aydin et al., 2019).
- Self-healing–Self-healing in biology has been explored and is being translated into bioinspired healing materials with the following mechanisms: protective coating, autogenous healing, shape memory, chemical activity, vascular systems, and bio-healing (Cremaldi and Bhushan, 2018). Bio-healing refers to the use of biological organisms to perform self-healing, such as spore-forming bacteria in self-healing concrete (Jonkers, 2007). Concrete also can self-heal cracks with water and carbon dioxide through chemical activity (Li and Yang, 2007). The crack closure of two different systems of self-healing concretes, based on polyurethane and superabsorbent polymers, has been successfully tested on large-scale prototypes (e.g., concrete beams of 150 mm × 250 mm × 3000 mm) (Van Tittelboom et al., 2016). Self-healing concrete has yet to be tested at the scale and environmental conditions of building foundations.

#### Root-Inspired Design Proposals for Building Foundations

Studying, abstracting, and transferring biological root system strategies to the field of foundation engineering can yield innovative designs, addressing the shortcomings of current foundation designs. In the following section, various bioinspired design strategies are presented at an abstract conceptual level, disregarding scaling and materiality at this point, which should be explored in further research projects.

##### Root-Inspired Erosion Prevention

**FIGURE 1:**
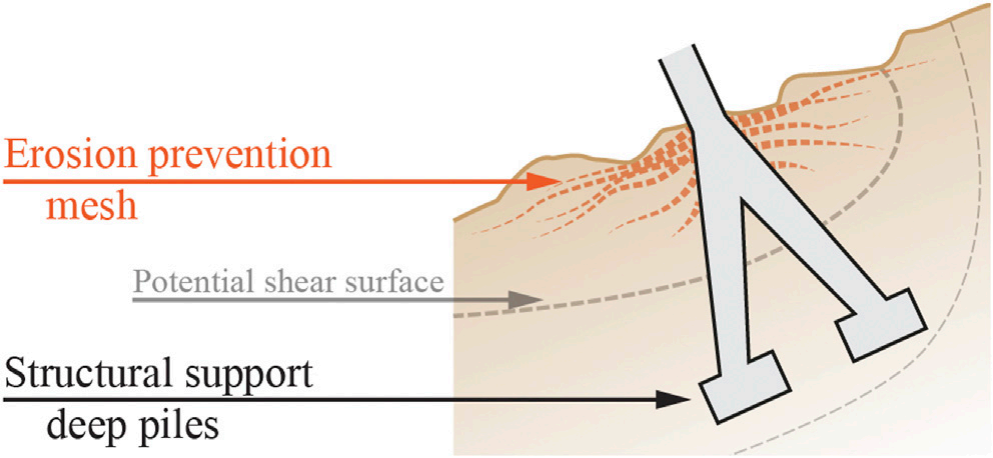
Soil retaining concept of a foundation on sloped terrain—The primary deep foundation piles support the structure beyond the potential shear surface and the secondary root-inspired network holds soil particles in place.

The following soil retention concept for a foundation is inspired by the erosion prevention of root systems in sloped terrains. Root systems have the capacity to prevent soil erosion by soil particle retention through entanglement and chemical bondage, but foundations are only designed to structurally support a structure (row 1 of Table 1). Foundations located in environments subject to soil erosion such as riverbanks, cliffs, or steep slopes would benefit from erosion prevention measures. The root system of a tree growing in sloped terrain develops vertical roots along with horizontal thin lateral roots retaining soil particles downslope of the tree (row 8 of Table 1). A technical building foundation could mimic this strategy and combine a main vertical structure to anchor the building with a secondary structure to retain soil particles (Figure 1). A mesh, similar to existing geosynthetic fabrics or a network of laterally branched elements near the surface, can be integrated in the design of foundations to reduce erosion. Soil particle retention could be achieved through chemical or mechanical attachment.

##### Root-Inspired Structural Support

The first concept of root-inspired structural support is inspired by the root grafting strategy found in forests to achieve cooperative building foundations (row 6 of Table 1). A newly constructed foundation can be connected to existing infrastructures to increase the load bearing area and the volume of soil recruited to support the structure, while making the foundation more resilient under extreme loading scenarios. Additionally, the connection to existing structures can provide an interface to exchange resources, such as water and thermal energy. The multifunctional aspect of this network of foundations is further described in section “**Multifunctional Root-Inspired Foundations**”.

**FIGURE 2:**
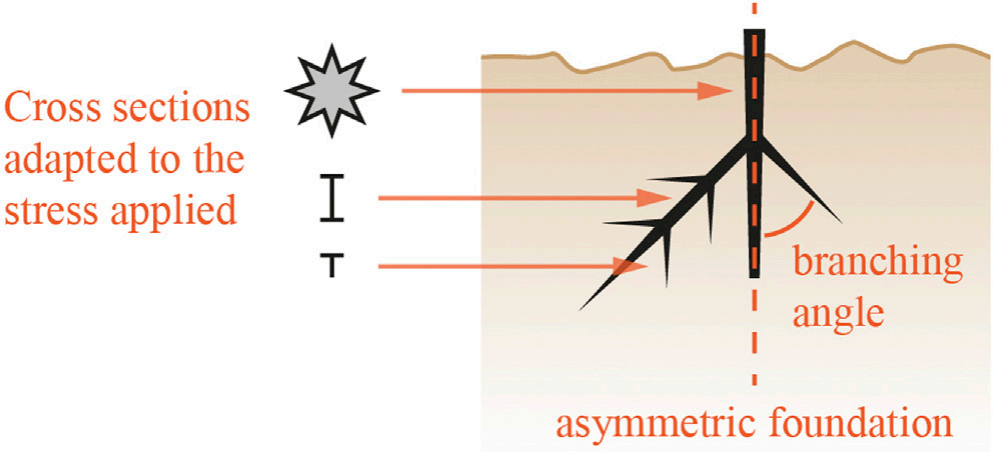
Structural optimization concept based on root adaptation and machine learning—The design of a root-inspired foundation follows the root traits adapted to a specific loading condition.

The second concept is on structural optimization based on both root adaptation to specific loading conditions as well as machine learning (row 7 and 12 of Table 1). Studying the adaptation of root systems to changing loads and environments can inform the design of root-inspired structural support systems subjected to similar loads. Computer simulations and machine learning can be used to process root adaptation data and apply the algorithms to foundation design. The following steps are required. First, root trait data about adaptation to various loading scenarios needs to be collected. A database will be populated with relevant traits in relation to the type of loads applied to the tree. A machine learning algorithm can then simulate how a root system would react to specific loading conditions. Finally, the morphology of this simulated root system could be used to inform the design of a new foundation (Figure 2).

The third concept of root-inspired structural support aims at translating the hierarchical structure of root systems for the transfer of structural loads to soil particles down to the microscale (row 13 of Table 1) through highly textured foundation surfaces. As this is interconnected with the soil insertion techniques, those concepts are explored in section “**Root-Inspired Soil Penetration Devices**”. Biological adhesives could also be secreted by the foundation to strengthen the bond between the foundation and soil particles as an analogy for mucilage (row 14 of Table 1).

##### Root-Inspired Soil Penetration Devices

In biology, multiple mechanisms allow organisms from animals to plants to move through granular media. The main question addressed in the following concepts is how to transfer biological strategies of root systems to an artificial soil penetrating system.

**FIGURE 3:**
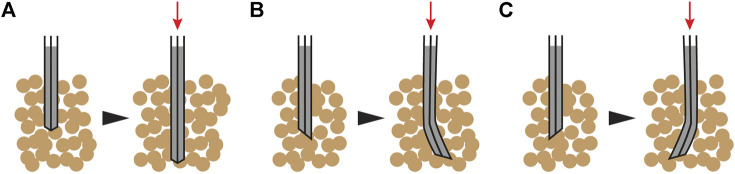
Foundation pile tips concept—When driven in the soil, a semi-flexible foundation pile with a symmetric tip remains straight **(A)**. In practice, soil particle arrangement will cause minor deflections depending on pile and soil properties. With an asymmetric tip, the same pile is expected to turn toward the acute side **(B,C)**.

The first concept of root-inspired soil penetration is on foundation pile tips inspired by the tapered root tip geometry that facilitates soil penetration (row 15 of Table 1). The tip geometry of a semi-flexible linear element affects its interaction with soil particles and the resulting path through soils during soil insertion. Therefore, controlling tip geometry could serve to guide a semi-flexible pile to follow a specific path (Figure 3).

**FIGURE 4:**
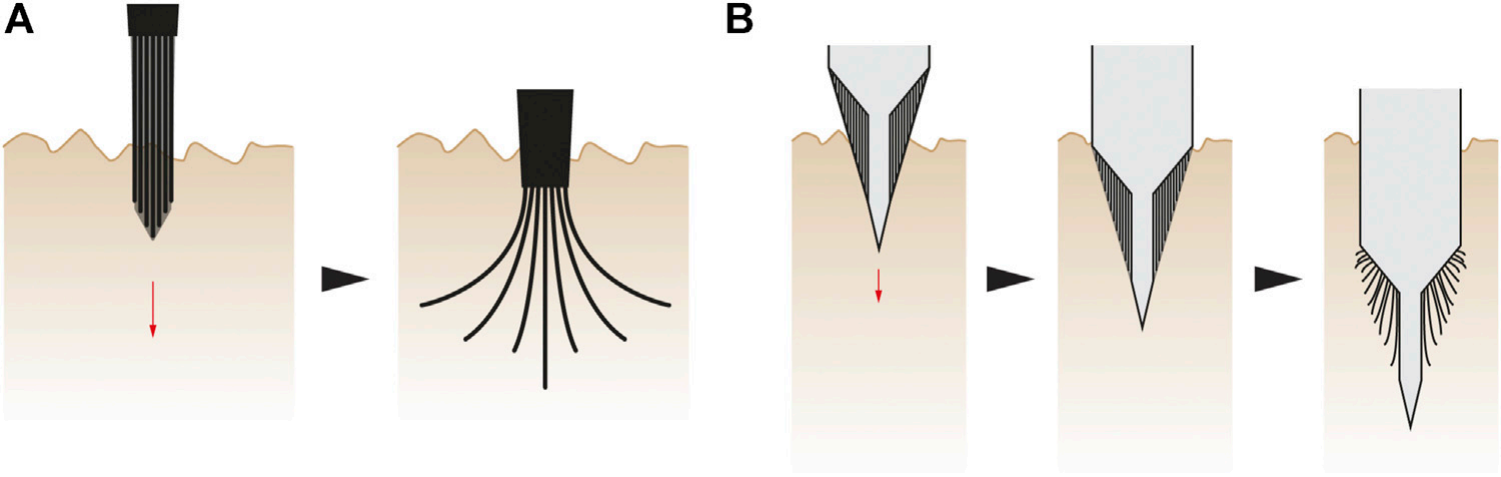
Branched foundation concept–This figure shows the application to the entire pile **(A)** or to parts of the tip **(B)**. When the pile is driven into the soil, the dissected elements follow different paths based on their geometry, flexibility and soil properties. The dissected elements can be controlled to reach a desired depth.

The second concept of root-inspired soil penetration is on branched foundations, emerging from the previous concept on tip geometry. First, the cross section of a pile made of semi-flexible material is extended into multiple thinner elements. Driving this dissected pile in the soil will produce a branched geometry that increases the load bearing area (row 5 of Table 1). The branched geometry is expected to be a result of the pile material properties, geometry of the dissected elements and their tips, and soil properties (Figure 4A). The tip geometry can also be actively controlled to distribute the branched structure throughout the soil in a specific arrangement. This concept of branched foundations can be applied to an entire pile tip (Figure 4A) or tip parts (Figure 4B).

**FIGURE 5:**
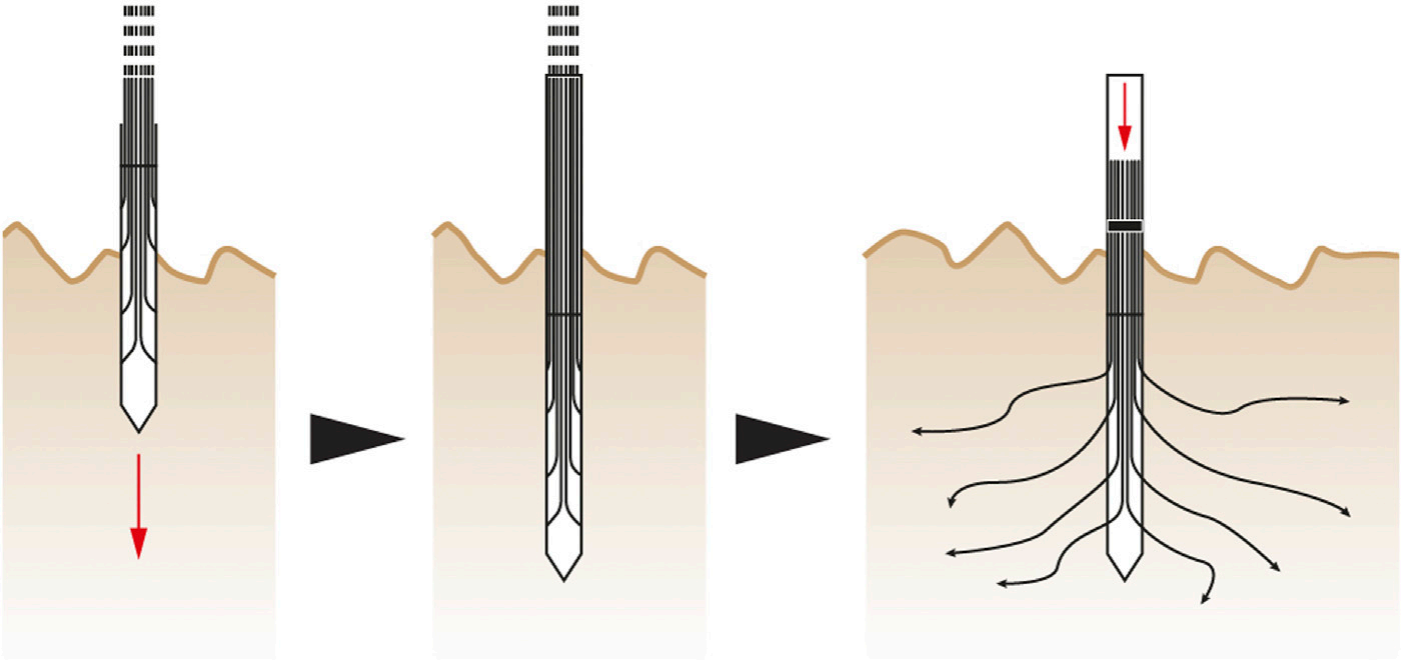
Hierarchical foundation concept—First, the smooth vertical pile is driven in the soil **(left)**. Once the pile is in place, individual semi-flexible elements are laterally pushed into the soil **(right)**.

The third concept of root-inspired soil penetration is on hierarchical foundations, based on the ability of roots to produce a complex branched structure in the soil through initial insertion of linear elements only. This strategy facilitates soil penetration while providing structural support at a later stage. Following this analogy, foundations can be designed for multi-phase implementation. A smooth linear vertical foundation pile can first be inserted in the ground. Thinner linear elements can then be pushed from this vertical pile into the soil laterally to improve anchorage (Figure 5). These lateral elements can also serve as anchors to push against while the foundation tip is driven deeper into the soil through axial expansion (row 18 of Table 1).

**FIGURE 6:**
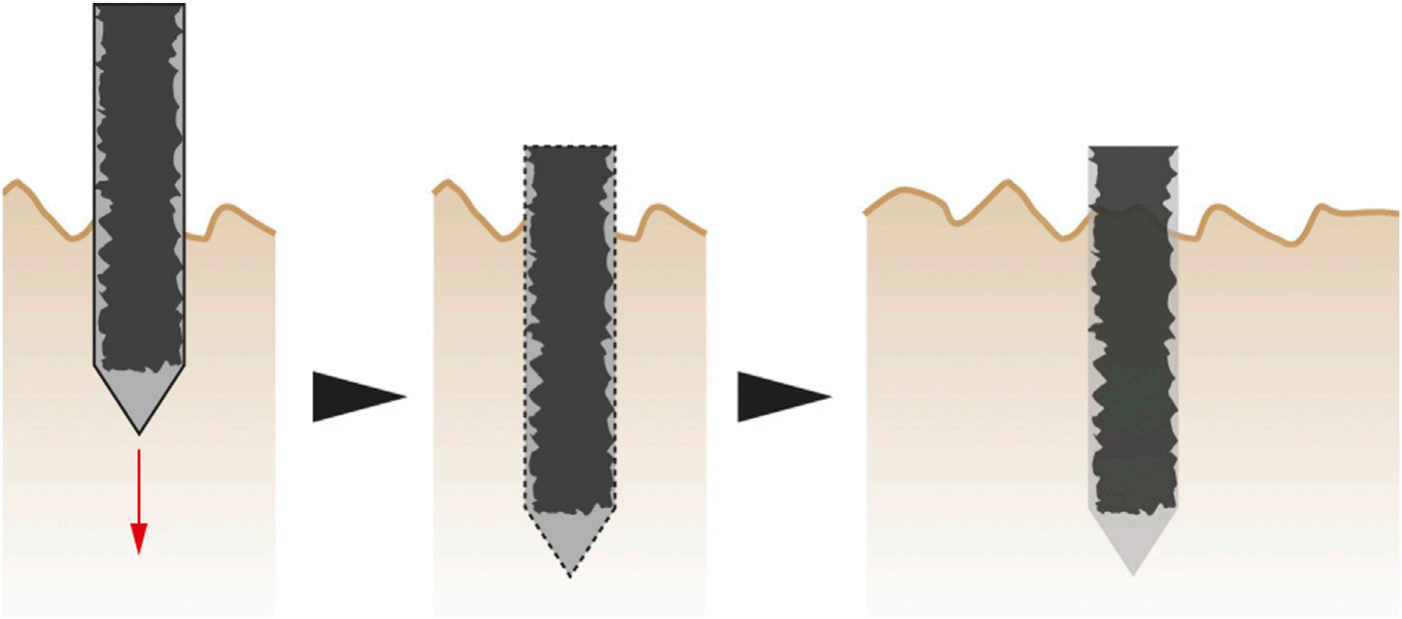
Texture alteration concept—First, the smooth foundation pile is driven into the soil **(left)**. Over time, the biodegradable material will decay **(middle)**, leaving the highly textured surface in contact with the soil particles **(right)**.

The fourth concept of root-inspired soil penetration devices is on texture alteration through material decay. Biodegradable material is placed around a highly textured foundation pile to create a smooth surface which facilitates soil penetration. Once inserted in the soil, this material will biodegrade and expose the highly textured surface, from the third concept of root-inspired structural support (“Root-Inspired Structural Support” section, row 13 of Table 1 and Figure 6). For this concept, additional bioinspiration of directional friction is interesting, especially if the directionality of the surface structure could change over time and by this, control the movement of the element through the soil. Bacteria known to precipitate calcium carbonate can be introduced under the biodegradable layer to further strengthen the bond between the foundation and soil particles.

Multiphase design was further conceptualized through the investigation of shape-change materials and structures for increased friction of foundation piles with weak soils, for example in wetlands (refer to the third concept of root-inspired structural support in “Root-Inspired Structural Support” section and row 13 of Table 1). Shape-change behaviors are used in three different concepts to counter the trade-off between the ease of pile insertion in soils and the surface friction of the pile.

**FIGURE 7:**
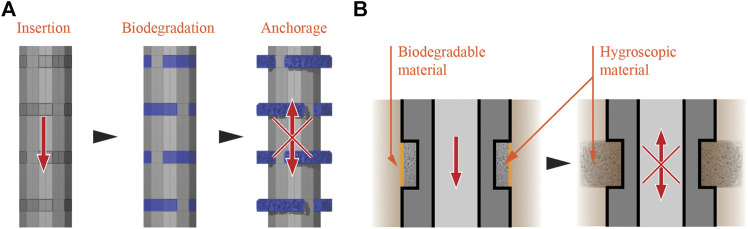
Shape-change foundation based on hygroscopic materials—The smooth foundation pile is driven into the soil **(A-left)**, then the biodegradable material decays **(A-middle)**. The material decomposition exposes the hygroscopic material to the saturated soil, resulting in a three-dimensional structure and increased anchorage through friction **(A-right), (B-left and right)** show the disposition of the materials before and after the decomposition of the biodegradable material.

The first concept of shape-change foundations is based on the swelling properties of hygroscopic materials when they absorb water. A hygroscopic material is located behind a biodegradable layer along the surface of a pile (Figure 7B). After placement in the soil and decomposition of the biodegradable layer, the hygroscopic material becomes exposed to water in wetland soil. The water triggers material expansion, creating a three-dimensional structure to increase the surface contact with soil (Figure 7).

**FIGURE 8:**
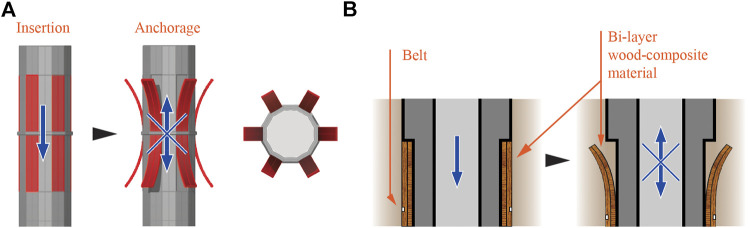
Shape-change foundation based on bi-layer materials—The smooth foundation pile is driven into the soil **(A-left)**. Over time, the bi-layer composite material, exposed to humidity, curves outwards resulting in increased anchorage through friction **(A-middle), (A-right)** presents a top view of this deployed pile system. **(B-left and right)** show the disposition of the bi-layer composite material and the belt holding it in place, before and after the curvature change.

The second concept of shape-change foundations is based on bi-layer materials, which change curvature under humidity gradients. Bi-layer plywood materials, inspired by pinecones, have been researched for their ability to bend under humidity gradients and applied to architectural prototypes (Menges and Reichert, 2015). Such composite material is located at the surface of the foundation pile. Once inserted in the soil, water absorption induces curvature change of the bilayer elements (Figure 8). The success of such shape-change concepts also depends on the resistance of the soil particles.

**FIGURE 9:**
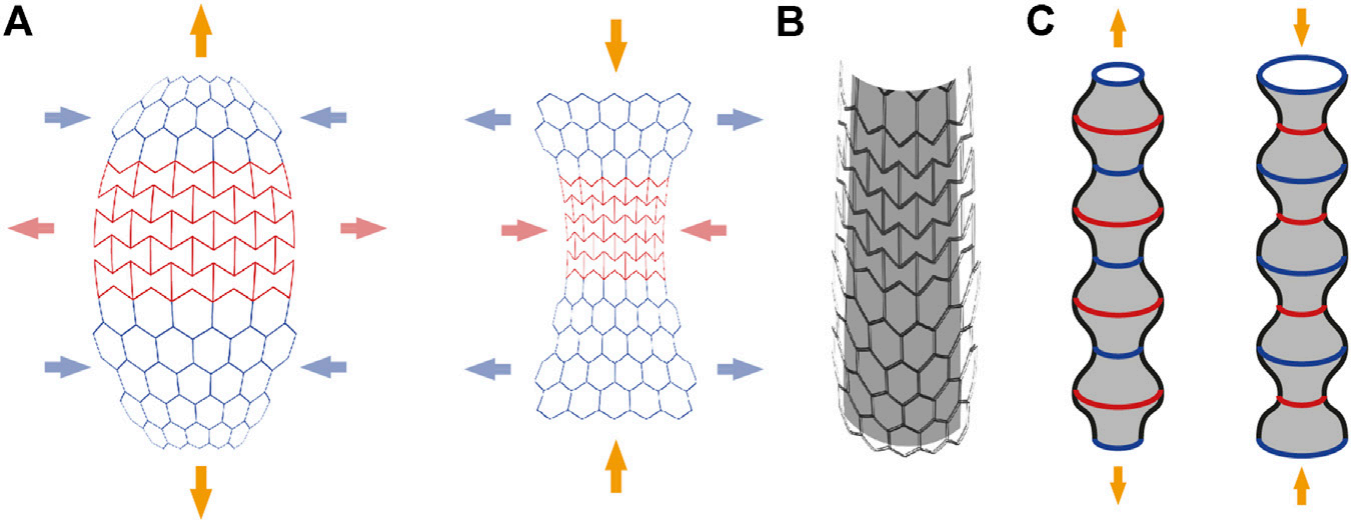
Shape-change foundation based on auxetic behavior—The combination of auxetic and non-auxetic structures in a plane produces edge curvature when compressed or stretched as simplified in **(A)**. When this combined structure is longitudinally stretched (i.e., yellow arrows), the auxetic section (i.e., in red) stretches while the non-auxetic one (i.e., in blue) shortens **(A-left)**. The reverse behavior happens when the combined structure is compressed longitudinally **(A-right)**. When rolled into a cylinder **(B)**, the longitudinal compression or stretching produces horizontal wrinkles **(C)**. This cylinder can serve as a vertical foundation pile to resist compression and tensile loads.

The third concept of shape-change foundations is based on the behavior of auxetic structures with a negative Poisson’s ratio. When stretched or compressed in one direction, they also respectively expand or compress in the perpendicular direction. By assembling auxetic and non-auxetic structures together in a plane, stretching of the assembly in one direction (see yellow arrows on Figure 9) induces a geometrical change of the structure (see red and blue arrows on Figure 9A) (Mirzaali et al., 2018). The assembly needs to be made of a semi-flexible material to allow material deformation. The flat assembly can be rolled to produce a cylindrical structure for a foundation pile (Figure 9B). During soil insertion, the structure can be locked and, once in place, released. Compressive or tensile loads on the auxetic foundation pile will create wrinkles leading to a higher bearing surface area (Figure 9C).

##### Multifunctional Root-Inspired Foundations

The multifunctional foundation concept is inspired by the added functionality in biological root systems and targets preventing erosion and exchanging energy and resources with the soil and other artificial structures (row 1 and 22 of Table 1). With the development of self-burrowing technologies and smart materials, multifunctional foundations can be envisioned. The benefits of erosion prevention have already been stated in “Root-Inspired Erosion Prevention” section. Foundations and geothermal systems abide by the same constraints of soil penetration and anchorage. Their combination into a multifunctional system could economize resources. Another further strategy to exchange thermal energy is to connect buildings through their foundations (refer to the cooperative concept in “Root-Inspired Structural Support” section). Appliances producing massive amounts of heat, such as data centers, can serve as a heat source for buildings (Woodruff et al., 2014). In addition to thermal energy, other resources such as water can be exchanged between buildings (row 6 of Table 1). By increasing load transfer through friction with the soil medium, the surface area of the foundations needs to be increased, but their weight can be decreased. As a result, hollow foundations can be a route for additional functionality, such as geothermal energy, water storage and transport.

The concepts presented in this section provide examples of how strategies found in root systems can inform the design of future foundations. These concepts do not take materiality, scaling, and rigorous technical feasibility into consideration however, but they should be the basis for future research and development projects.

### Coastal Infrastructure

#### Current Coastal Infrastructure Design and Problem Review

Typical built coastal infrastructure serves two main objectives: protection from wave action and landward erosion (USACE and Army Engineer Waterways Experiment Station Coastal Engineering Research Center, 1984). While generally effective at these objectives, coastal structures are static, often anchored, and therefore cannot adapt to rapid, dynamic conditions. In light of climate change, current static structures do not hold up to raising water levels, storm surges, and flooding. For example, a post Hurricane Katrina rebuild of the New Orleans, LA, USA seawall was almost overtopped by waves from storm surge in 2018.^2^ Additionally, hard infrastructure alters and displaces the structure and function of natural habitats that existed before, eliminating both significant biodiversity, and habitat complexity that supports trophic structure development for a rich, interconnected food web, refuge for mobile organisms and fish, and attachment surfaces for sessile and habitat-forming organisms (Strain et al., 2018).

Common typologies of hardened infrastructure include: shore-parallel attached smooth vertical or concave surfaces (e.g., seawalls, such as bulkheads), shore-parallel attached sloped variable surfaces (e.g., revetments, such as riprap), shore-perpendicular attachments (e.g., groins and jetties), detached shore-parallel sloped above-water structures (e.g., breakwaters), and detached shore-parallel submerged structures (e.g., breakwaters and artificial reefs). Shore-parallel attached structures prevent erosion of land from wave action but fragment the land-water interface and contribute to the loss of natural habitats (Goodsell et al., 2007). Seawalls and some revetments reflect waves, which increases nearshore turbulence (Silvester, 1972). Often this turbulence is too rough for native plants to establish and maintain, attracting invasive species establishment. Increased turbulence also increases sediment resuspension and reduces water clarity.^3^ Depending on wave action and nearshore particle size, sediment may be carried through wave reflection out into the open shore, reducing the available sediment budget for natural littoral deposition processes. Riprap revetments can fail due to toe scour, outflanking, wave overtopping and subsequent erosion of material behind the revetment, and settlement. Wave reflection also causes scour, deepening the water level adjacent to a seawall, allowing for larger wave heights to approach the shore (Griggs and Fulton-Bennett, 1988). Shore-perpendicular attached structures redirect littoral transport to prevent erosion or allow river mouths to remain deep enough for navigation in the case of harbor infrastructure, but often cause downdrift erosion due to a reduced available sediment budget for continued nearshore transport. This disruption of natural littoral processes induces a negative feedback loop, requiring more downstream infrastructure to protect against this erosion (Hanson and Lindh, 1993). Both detached above-water and submerged structures attenuate waves (through surface wave breaking and bottom roughness, respectively) and provide fish habitat, but above-water structures restrict coastlines from migrating landward or seaward in response to varying water levels (McLachlan and Defeo, 2018; Scape/Landscape Architecture PLLC, 2014).

Mangrove forests show a pathway to remediate these shortcomings. Mangrove roots stabilize soils, while their ecosystem provides habitat and a gradual land-water transition. On a long time scale, mangrove forests migrate landward or seaward in response to varying water levels (Robertson and Alongi, 1992). Wave dissipation through these complex flow obstruction configurations significantly reduces wave reflection and subsequent turbulence in the nearshore environment. Even if mangroves are overtopped by waves during storm surge, the roots and trees still provide adequate bottom roughness and flow obstruction to effectively attenuate wave energies (Mazda et al., 1997). Manmade constructions using wood, such as rootwad revetments, engineered log jams, crib-walls, deflectors, weirs and pile dikes, also stabilize soils, reduce flows, while also providing habitat and maintaining a more gradual land-water transition. Interestingly, while these structures are cheaper, exceed project design life, and often match or exceed performance objectives compared to rock structures, these LWD human constructions are rarely used (Abbe et al., 1997). Additionally, mangrove roots, naturally occurring log jams, and woody overhang along riverbanks or shorelines, provide habitat for a variety of organisms. Complex morphologies, such as root systems, protect from wave action and stabilize sediment to primarily provide anchorage for an aboveground structure, as well as provide habitat. Complex morphologies would similarly allow for multifunctional coastal infrastructure design.

To undertake a redesign of coastal infrastructure that expands beyond its primary objectives of protection from wave action and landward erosion, a biomimetic approach via the study and abstraction of root systems can be employed. Investigating specific themes of erosion prevention, multifunctionality, spatial variability, and adaptation to dynamic external loads involves answering the following research questions: What is the minimum level of complexity required from a root-inspired structure (e.g., topology, orientation of elements, density, distribution of individual cross-sections across topological orders, distribution of orientation across topological orders, texture) and at what scale(s) is it most effective for the following functions of (a) wave dissipation and dispersion (vs. wave reflection), (b) downstream development of reduced flow velocities through the depth of the water column that match preferential velocity ranges of native taxa, (c) flow speeds for sediment deposition, and (d) refuge/pore space habitat creation? What minimum combination of effective length scales as informed by root systems are needed to discourage localized erosion and scour development by dissipating the formation of vortices? Furthermore, can topographically complex infrastructure be produced that more closely resembles the structure and function of the natural habitat (such as woody overhang, exposed root systems) that has been displaced? With regards to adaptation and multifunctionality, coastal infrastructure of the future should adapt to changing external loads such as wave height, storm surge, sediment movement, or landslides in sloped banks or shorelines. Coastal infrastructure should also participate in additional ecosystem services such as provision of habitats, nutrient cycling, and carbon sequestration.

#### Current Innovative Solutions for Coastal Infrastructure Design

In the following list, we summarize current alternative approaches to traditional engineered coastal infrastructure design, spanning complex forms, coastal ecosystem restoration, to living infrastructure. They are organized under four main topics of interest referring to the analogy table in “Abstraction and Analogy” section (Table 1): soil erosion, structural support, conditions for living organisms, and multifunctionality.

- Geo- and bio-textile fabrics to prevent soil erosion–As mentioned in “Current Innovative Solutions for Foundation Design” section, geosynthetic products are currently used to stabilize soils through placement of a polymeric textile filament layer by layer around loose rocks, gravel, or sediment. This practice is also seen in coastal engineering. Geotextile tubes or bags, a synthetic fabric filled with sediment, are used to line riverbanks, shorelines, or protect young plant seedlings as part of a nearshore ecological restoration initiative. Biodegradable coconut coir pith logs packed in tubular netting, known as coir logs, are an example of soil bioengineering that reduce water velocities at the edge of slopes, shorelines, and riverbanks (Rella and Miller, 2012).
- Complex concrete forms for increased structural support—Concrete forms for revetments, breakwaters, or additional reinforcement of seawalls have become more complex since the 1950s with inventions such as Tetrapods, Akmons, Seabees, Accropodes, Xblocs, dolos, and KOLOS. Their complex shapes, pack density, and porosity allow for wave dissipation that reduces wave run-up, overtopping and reflection, but also facilitates interlocking of individual units and increased stability of the overall structure (Dupray and Roberts, 2009).
- Establishing conditions for living organisms through ecosystem conservation and restoration—Wetlands, mangroves, coral reefs, oyster reefs, and salt marshes are proving cheaper and more effective in reducing wave energy than building hard artificial structures. Meta-analysis of the literature indicates that coral reefs reduce wave heights by 70%, salt marshes by 72%, mangroves by 31%, and seagrass/kelp beds by 36% (Ferrario et al., 2014; Narayan et al., 2016).
- Establishing conditions for living organisms through eco-engineering—Locations such as harbors, nearshore navigation routes, and dense urban areas are not suitable for restoration. In this case, ecological engineering or “eco-engineering” is an approach that considers recovery of prior ecosystem services in the design of hard infrastructure (Mayer-Pinto et al., 2017). Habitat features to increase fish productivity or biodiversity of key functional groups of organisms can be integrated via textures, crevices, pits, intertidal water retaining features, raises, ledges, ridges, and soft, flexible protruding elements such as rope, ribbon, or twine (Strain et al., 2018). Grooves, dimples, and grooved shelf features were incorporated into the submerged toe blocks of offshore breakwaters in Lake Erie, part of the Great Lakes freshwater system, to increase habitat for fish and invertebrates with limited success (Suedel et al., 2016).
- Multifunctional, living infrastructure—ECOncrete uses a special concrete mix to lower the pH closer to that of seawater, a common criticism of traditional marine grade concrete, to facilitate organism attachment and growth (Finkel and Ido, 2017). The concrete blocks are formed with molds to create the surface texture and roughness to promote attachment by oysters, bryozoans, coralline algae, and several other habitat-forming species (Perkol-Finkel and Sella, 2015). Uses include offshore breakwaters, revetments, seawall panels, or attachments to existing seawall panels.^4^ Reef Design Lab 3D prints unique surface features on seawall panels using marine grade concrete to improve recreational fishing opportunities and increase biodiversity, specifically to maximize colonization of native species.^5^ Mangrove Reef Wall was first studied to understand flow-structure development behind modeled mangrove roots, as well as wave attenuation and sediment deposition characteristics to create bioinspired infrastructure (Kazemi et al., 2017). The current application of this research is a living seawall application for wave attenuation, colonization, and increased biodiversity.
- Multifunctionality of hard infrastructure to assist with coastal restoration and rehabilitation—A wide range of coastal restoration and rehabilitation projects use hard modular structures from concrete mixtures. TetraPOT, by designer Sheng-Hung Lee at National Cheng Kung University, creates an interlocking system of concrete pods that use mangrove trees and roots to keep the pods in place as a line of coastal defense along shorelines.^6^ Reef Design Lab takes a similar approach with a reusable planter to promote mass planting of a native mangrove species for coastal defense.^7^ CEMEX created the Rhizolith Island (“Isla Rhizolith”) prototype, consisting of a mosaic of floating concrete structures with a “head” and a “fin” that functions as a seed carrier for mangroves, to restore mangrove forests while also providing coastal protection. The fin is also designed to serve as marine habitat, offering shelter for fish and surfaces for barnacles.^8^ Reef Ball also uses a specialized concrete mixture to lower the pH and a textured outer surface to promote growth of transplanted corals. Reef Ball uses similar principles to develop concrete domes to serve as oyster beds for oyster reef restoration. Used in more than 70 countries, on more than 4,000 projects, there are more than 700,000 Reef Balls in the oceans around the world.^9^
- Complex scaffolding to establish conditions for living organisms—Grow Oyster Reefs LLC has created complex concrete scaffolds mimicking the oyster shape, in addition to mimicking the oyster shell’s material formula through a calcium-enriched, patent-pending mixture that also aims to reduce nitrogen levels in water.^10^ Additionally, Reef Design Lab 3D prints ceramic scaffolds using D-shape technology to assist with coral reef rehabilitation.^5^ These two examples show the possibility of production of complex coastal structures.

#### Root-Inspired Design Proposals for Coastal Infrastructure

In this section, several bioinspired design strategies from the biology of root systems are presented for coastal engineering. Since these strategies do not depend on the availability of real mangrove trees, riparian tree species, or rootwads, the properties of these root-inspired structures can be fine-tuned according to the learnings from biomechanics investigations. Parameters like the distribution of cross-sections, lengths, spacing, branching angles, and orientations, can be adjusted to a specific shoreline reach with its predominant wave and storm surge conditions. Additionally, the arrangement, stacking, and orientation of several root-inspired structures can be adjusted for different shoreline configurations and wave energy conditions, as well as intended ecosystem service restoration goals and outcomes, and/or maintenance strategies.

##### Root-Inspired Erosion Prevention

**FIGURE 10:**
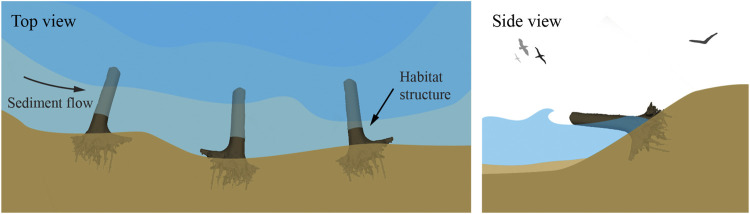
Erosion prevention concept along a shoreline—The top view **(left)** shows modeled rootwads embedded along a shoreline with root fan facing landward and truncated trunk (note transparency in open water) facing seaward. The side view **(right)** shows one rootwad with root fan embedded along a sloped shoreline face.

The first erosion prevention concept builds upon the concept of engineered log jams and complexes discussed in “Root Utilization in Human Constructions” section (row 3 in Table 1), a windthrown tree overhang along a river or stream still embedded in the bank by its root system, and mangrove roots encouraging sediment deposition (row 4 in Table 1). If erosion is of highest concern, a root-inspired structure (or several structures) can be inserted perpendicular to a beach or shoreline face with the root fan embedded in the shoreline (Figure 10). The multi-scale elements of the root-inspired structure, such as overall shape, topological orders, and branching angle/orientation, will need to be tested to determine their effects on vortex development, localized erosion, and scour, so as not to be a further detriment to the shoreline. These multi-scale elements could be engineered such that vortices do not form (or are quickly dissipated) behind or downstream from the structure, further enhancing sediment deposition potential. Additionally, since groins and jetties cause downstream erosion issues due to a perpendicular element facing seaward into the nearshore, the seaward end of a root-inspired structure can be truncated so as not to cause similar issues. This truncation is shown in Figure 10 (left) as the transparent ends of the trunk of a 3D modeled rootwad. This seaward end could then be formed to provide heterogeneous substrate for habitat. Sediment penetration of the complex root fan like end of this structure into the shoreline face may be difficult. Concepts to increase contact area with the sediment previously described in “Root-Inspired Soil Penetration Devices” section on building foundations could be employed.

This erosion prevention concept could also be utilized particularly during high water years to protect shoreline property. Depending on sediment type (silt, clay, sand, mud), particle size distribution, and wave energy exposure, this concept could unintentionally cause localized scour around the large anchoring elements (Svoboda and Russell, 2011) and would need to be tested to confirm its effects. Additionally, several engineered log jams or complexes, such as bar apex jams (“Root Utilization in Human Constructions” section), could potentially be embedded in beach sediment at different distances from the water line to provide erosion protection of the entire beach front. The complexes can be designed more as a fixed structure, but may still more closely mimic the process of both large driftwood and windthrown trees near a historically forested shoreline forming natural protective “structures” along a beach (Abbe et al., 1997).

**FIGURE 11:**
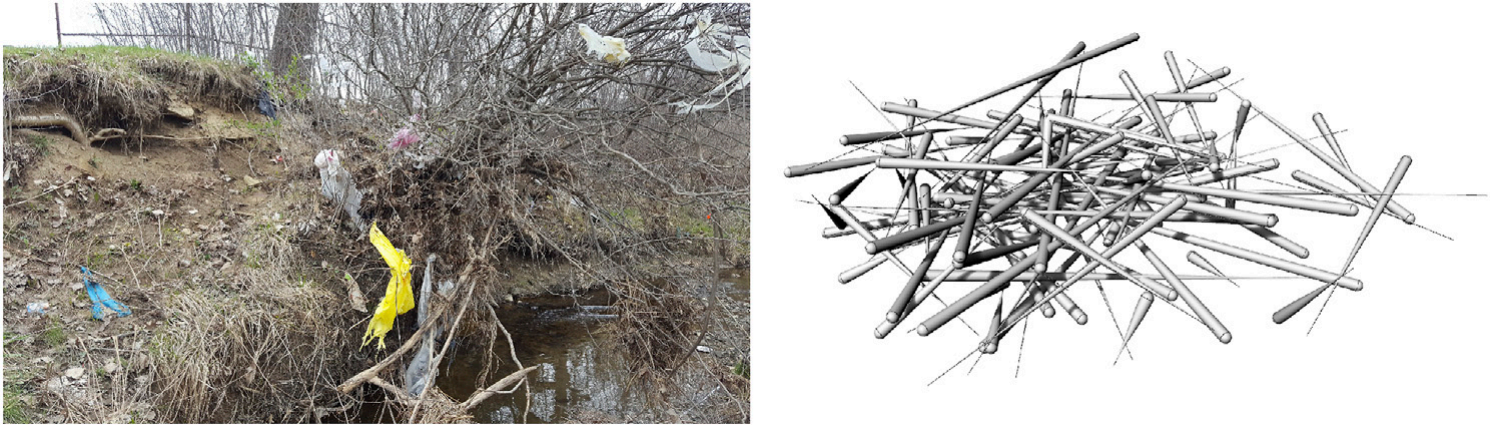
Erosion prevention concept for engineering structures—A typical exposed root system along a riverbank catching plastic debris **(left)** can be one of several functions of an abstracted root structure **(right)** that could replace the ends of bridge abutments or the edges of culverts to reduce erosion and scour.

The second erosion prevention concept specifically addresses additional engineered structures in or near waterways that can cause significant erosion issues. This includes structures such as bridge abutments and culverts, not primarily used for erosion prevention of coasts, streams, or riverbanks, that cause localized scour or erosion at the edge or slightly downstream of the structure. These engineered structures could be redesigned based on the geometry of root systems (Figure 11—right), in order to reduce localized scour and erosion and additionally deposit sediments further downstream that are a result of scour or erosion. When implemented, rootwad-inspired structures will also catch plastic waste (Figure 11—left) that could be collected at regular intervals, to reduce overall transport of waste to lakes and oceans. The same structure placed strategically at the bottom of a river could reduce bedload movement and scour.

##### Root-Inspired Multifunctional Revetments

**FIGURE 12:**
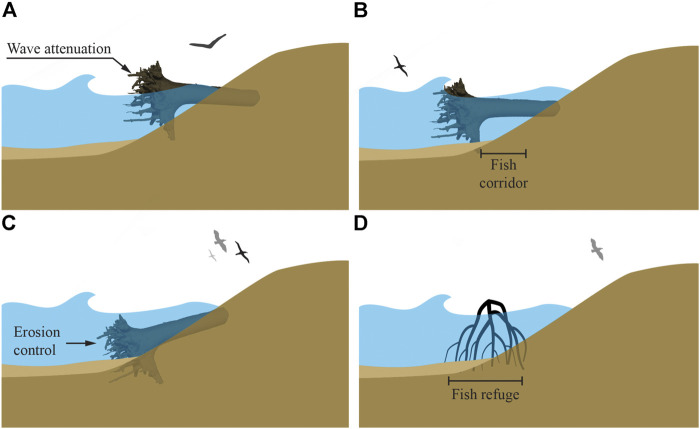
Multifunctional revetment concept designs—**(A)**—A modeled rootwad illustrating wave attenuation with the root fan facing seaward, and the trunk end embedded along the sloped shoreline face. **(B)**—Spacing between the root fan and sloped shoreline face shows possible passage for a fish corridor. **(C)**—A root fan embedded more in the sediment bottom may provide greater toe protection of a steeper shoreline face. **(D)**—A mangrove like structure can attenuate waves in addition to providing habitat (fish refuge) through spacing control.

Multifunctional revetment design concepts are additional iterations of the concept described in Figure 10, but with the root fan facing seaward. This design concept (Figure 12) is different from current wooden revetments in that there is a complex flow obstructing end, but similar to riverbank stabilization practices used in restoration ecology (row 2 in Table 1 and “Root Utilization in Human Constructions” section). One purpose of the root fan like end is wave attenuation, breaking up and dissipating the waves due to the density, orientation, and cross-section of the individual elements in the structure (Figure 12A). Wave attenuation also in turn reduces erosion potential. The spacing between the root fan and shoreline face can be manipulated, creating space for a protected fish corridor or passage behind the root fan, a slower moving wake region for aquatic plants to establish and maintain, and/or the ability for the shoreline to migrate landward or seaward (Figure 12B). This sub-strategy tackles the larger theme of creating conditions for living organisms (rows 20–21 in Table 1.) Shipping and navigational activities, recreational activities, and predominant wave conditions may restrict available space. The root fan can also be angled down and embedded into the sediment bottom, offering more traditional toe protection for a sloped shoreline face in addition to habitat, dependent on wave conditions (Figure 12C). Mangrove like revetment structures could be adapted to provide habitats (rows 20–21 in Table 1), by controlling spacing between individual elements of a single structure (Figure 12D).

##### Root-Inspired Multifunctional Composite Structure

**FIGURE 13:**
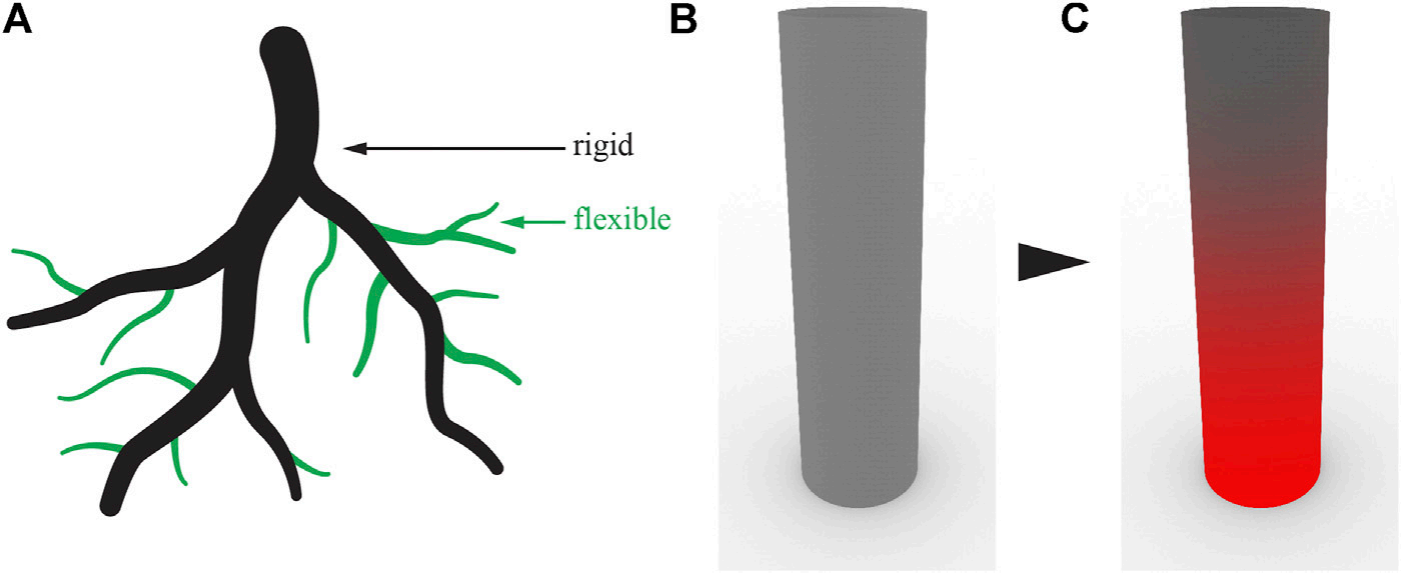
Multifunctional composites—**(A)**—A root-like structure with the black elements comprising a rigid material and the green elements comprising a flexible material. A standard construction pile made of one material is shown in **(B)** while an innovative composite pile is shown in **(C)**, composed of a material gradient from more rigid (red) at the base of the pile to more flexible (grey) at the top of the pile.

Two design concepts shown in Figure 13 illustrate the same principle idea: use of multifunctional material composites. With the advent of additive manufacturing, even in using traditional coastal construction materials such as marine grade concrete and ceramic (“Current Innovative Solutions for Coastal Infrastructure Design” section), material composites reveal new possibilities in multifunctional infrastructure. Building on the fact that roots have different functions and respective morphology, in addition to the morphological adaptation principles illustrated in rows 7–9 of Table 1, material properties could be varied. A composite structure may employ more rigid, thicker material allocation in places exposed to wave energy and erosion potential (i.e., higher stress), while softer, more flexible material could be allocated in sheltered orientations for habitat or refuge. Figure 13A shows this division in material rigidity and flexibility in a root-inspired structure, while Figure 13B shows a gradient in material rigidity in a standard pile. As previously mentioned in Kazemi et al. (2017), flexibility of a modeled mangrove root resulted in higher drag in shallow waters; therefore, flexibility along the axis of a standard structural pile may offer greater flow reduction in some lower flow scenarios than a standard rigid pile. A structure modeled after an engineered log jam could also have both rigid and flexible elements assembled in one continuous, porous, yet stable structure (row 3 and 4 in Table 1).

##### Root-Inspired Patterning for Multifunctional Seawall

**FIGURE 14:**
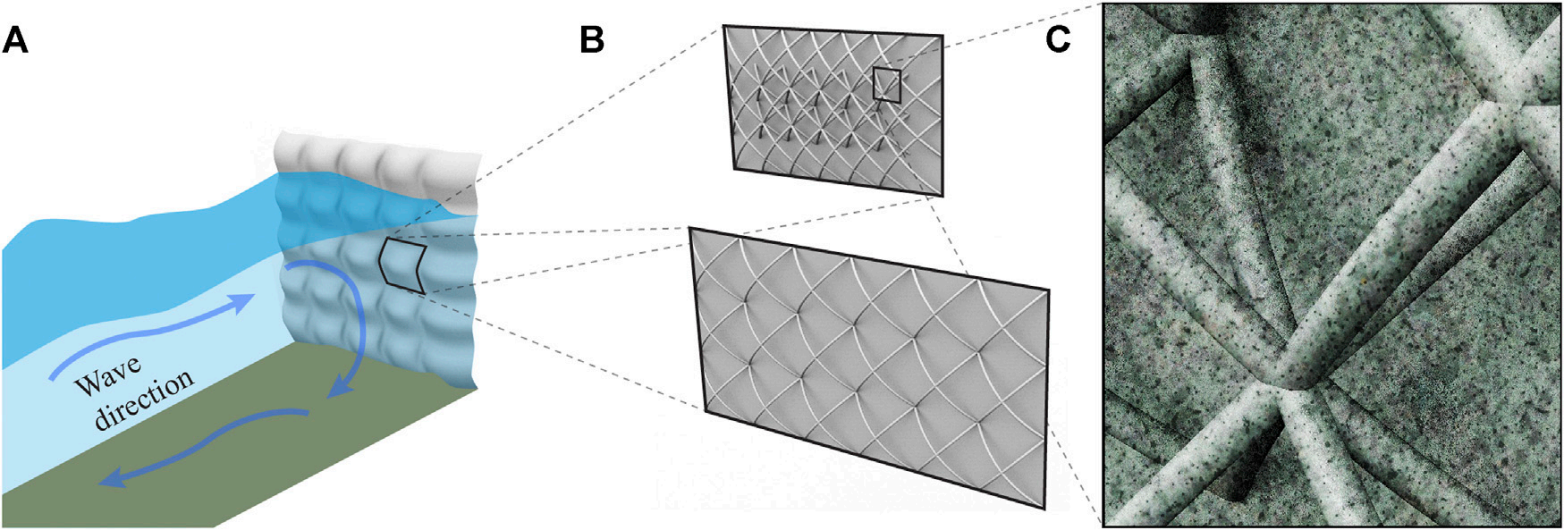
Multifunctional seawall patterning—**(A)**—Large-scale undulations on the entire face of the seawall so that it is no purely vertical, and no longer causing wave reflection and scour as shown with the blue arrows. **(B)**—A simple fractal pattern on an individual seawall tile. **(C)**—Magnification of an individual seawall tile illustrating surface roughness and texture.

This concept suggests a large-scale redesign of a seawall, including micro and macro approaches. Building on the existing living seawall innovations described in “Current Innovative Solutions for Coastal Infrastructure Design” section, a seawall could have large-scale undulations on the entire face rather than just at the top and bottom like a recurved seawall. The hypothesis is that this large-scale undulation (Figure 14A) would significantly reduce wave reflection and subsequent toe scour compared to a recurved seawall. Figure 14B shows a seawall concept with a hierarchical surface design. The designs of Reef Design Lab and Mangrove Reef Wall (“Current Innovative Solutions for Coastal Infrastructure Design” section) could also be utilized at this scale. These existing designs offer spatial variability, referring to horizontally heterogeneous and topographically complex structures and surfaces typically observed in natural habitats. Figures 14–C magnifies the surface roughness and texture, building on the habitat utility of snag/root roughness as described in row 21 of Table 1. While a seawall does not mimic a root system in any tangible abstract way, the concept of irregularity (row 4 of Table 1), root curvature, spacing, and morphology can be integrated by the application of two-dimensional patterns or three-dimensional surface structures.

The concepts presented in this section provide an overview of how strategies found in root systems can inform the design of technical coastal infrastructure. These concepts do not take into consideration however, materiality, scaling, and rigorous technical feasibility, which could be further researched in future projects.

## Discussion

This paper demonstrates utility of the bioinspired design approach through the study of the biology of root systems to inform multiple engineering design applications. Through design of a comprehensive analogy table that relates specific biological information about roots to engineering infrastructure problems and vulnerabilities, functional principles were established to link the two fields as outlined specifically through the biomimetic process. These principles informed design proposals for foundation and coastal engineering that can fulfill various functions, such as erosion prevention, structural support, soil penetration, and habitat creation. Many questions emerging from this work are not addressed in this paper however, specifically in the areas of materiality, technology, sustainability, and implementation.

Considering materiality, typical foundation and coastal engineering constructions use wood, concrete, rock, and steel. The resources required to shape these traditional materials and their desired material properties result in simple morphologies. New techniques such as 3D/4D printing, dual-extrusion, D-shape technology, and CNC machining, allow for customizable and complex organic forms, such as root-inspired structures. Field scanning techniques, parametric design, and advanced manufacturing techniques could be combined into a unified design process to customize structures to specific site conditions and desired functions. Traditional engineering materials can be shaped with these new technologies, but in parallel, such technologies foster the exploration of a wide range of material composites. Engineered material composites can be highly tuned with specific properties and performance characteristics to potentially respond and adapt to dynamic loading conditions. A material’s engineered response at smaller scales (i.e., micro- and nano-level) is akin to biomass accumulation in locations of higher stress in trees. Added functionality, complexity, and feedback loops through the developing fields of biotechnology and synthetic biology can also be considered in subsequent design iterations. The use of living organisms (e.g., mycelium, coral polyps, oyster spat) and modified living organisms can lead to emerging techniques like MICP (Dade-Robertson et al., 2018). To abstract root systems principles for foundation and coastal engineering, the transfer of different timescales needs to be addressed in further research. Damping systems, responsive MICP, and self-healing materials could respond to everyday fluctuations. Digging/growing agents, programmable structural growth, and design flexibility to repurpose infrastructure meanwhile, could serve as an adaptation to long-term loads.

Advanced technologies and materials could lead the way to adaptable engineered systems. In foundation design, we can envision adaptation through material or shape change response to changing soil conditions, changing structural loads throughout the lifetime or utility of the structure, or to strengthen the foundation over time (similar to secondary thickening in root systems). In coastal infrastructure design, we can envision adaptation to the changing energetics of nearshore systems, water levels, nutrient or pollutant concentrations (e.g., material surface properties facilitating in removal or sequestration), and/or dissolved oxygen provisioning for aquatic life. For achieving sustainability, the design of a product should be evaluated for its entire life cycle, which cannot be performed at this early design stage. Therefore, concepts presented in this paper focus on the primary functions required and opportunities for improving existing practices toward greater sustainability, a key aspect of biomimicry. Assessing the sustainability of these concepts would need to question and include the longevity, adaptation, decay, degradation, and/or reusability of such systems. Should elements of foundation and coastal systems naturally decompose or degrade in the ground or water, or should they be reusable or recyclable? Does the design for adaptation to changing conditions over time increase design complexity to the point where it may lead to reduced sustainability? For material selection, biological-based fillers originating from agricultural or construction waste streams can be utilized in material composites. Especially in coastal engineering, degradation of these inert biological-based fillers in an engineered material composite is an additional component to consider, whether by saltwater intrusion, ice, or UV light. Degradation could be seen as beneficial, considering if the by-products offer a food source for native organisms, do not disrupt organismal primary productivity and reproductive cycles, and if habitat-forming organisms may take the place of the degrading structure over time. Must the coastal structure be permanent, or once the desired physical conditions are established (i.e., sediment deposition, coastal vegetation fully established to reduce wave heights), the structure becomes indistinguishable from its surroundings? Similarly, the life cycle of a building foundation could be designed such that it decomposes or “dies” (similar to lateral roots or root hairs no longer needed for water and nutrient acquisition) when the building is no longer occupied. The foundation could also connect to the soil matrix at the individual soil particle scale to continue preventing erosion, even though aboveground structural support may no longer be needed.

Lastly, in the case of implementation, where do bioinspired design concepts of built infrastructure fit in the existing array of technical options? The possibility to reuse, retrofit, or recycle existing foundations should be a priority to reduce waste production and urban decay. Instead of following the ‘take-make-dispose’ linear process in building construction, technological advancements allow for analysis and adaptation of existing structures to current needs, instead of building new structures to fit new needs. Future designs must follow a more integrated approach, “managing engineered landscapes as ecological systems,” that evolve, adapt, and respond through time (DeJong et al., 2015). Additionally, root-inspired structures should not replace necessary hard coastal infrastructure in high energy nearshore systems where it is required nor restoration of ecosystems in low energy systems where it is possible. Their inclusion may offer additional functionality or allow for conditions for successful ecosystem restoration to take place in systems where these projects typically cannot succeed.

## Conclusion

The design of built infrastructure often regards soil properties as stable through time. By default, building foundations to seawalls are both bulky and heavy to respond to predominant loads and to ensure stability and durability over a long lifetime. Dynamic changes to soil properties and environmental conditions, in addition to inefficient use of material and poorly optimized construction by viewing soil as stable, compromises built infrastructure performance.

While foundation designs are limited to simple vertical geometries by current building techniques, diverse strategies from root systems give insights to develop multifunctional foundations able to anchor structures, prevent erosion, and adapt to various stresses. Several conceptual designs were made by abstracting and combining multiple root strategies relating to adaptive soil penetration, surface texture, complex topology, hierarchical morphology, self-healing materials, and growth principles. Similarly, coastal infrastructure is often limited to two technical objectives, simplifying form, material, construction, and implementation, which displaces natural habitat and exacerbates negative feedback loops in coastal ecosystem functioning. Strategies adapted from root systems, and in particular the ecosystems supported by mangrove and other coastal forests, can serve to develop multifunctional coastal infrastructure. In particular, principles relating to root system architecture, surface texture, complex topology, material gradients, and adaptive soil penetration were abstracted and combined into several conceptual coastal infrastructure designs.

We conclude that bioinspired design concepts of built infrastructure should be part of the mosaic of solutions offered that provide protective, multifunctional, and livable spaces. Therefore, this review of biological root systems and the conceptualized biomimetic translations offers a new way of thinking about technical problems and vulnerabilities in engineering and broadly contributes to creating an improved understanding and intersection of the fields of biology and engineering.

## Footnotes

1
https://selfassemblylab.mit.edu/rock-printing/

2
https://www.nytimes.com/interactive/2018/02/24/us/new-orleans-flood-walls-hurricanes.html

3
https://www.mishorelinepartnership.org/erosion-at-the-shoreline.html

4
https://econcretetech.com/

5
https://www.reefdesignlab.com/

6
https://www.jamesdysonaward.org/en-GB/2016/project/tetrapot/

7
https://www.reefdesignlab.com/mangroveplanters

8
https://www.cemex.com/-/cemex-develops-floating-concrete-island-to-revitalize-mangrove-shorelines

9
https://www.eternalreefs.com/the-eternal-reefs-story/about-reef-balls/

10
http://www.growoysterreefs.com/
